# The attitudes of hospital directors towards normalising accreditation standards: A qualitative descriptive study for Saudi Arabia

**DOI:** 10.1093/intqhc/mzac070

**Published:** 2022-09-01

**Authors:** Mohammed Hussein, Milena Pavlova, Wim Groot

**Affiliations:** Department of Health Services Research, CAPHRI, Maastricht University Medical Center, Faculty of Health, Medicine and Life Sciences, Maastricht University, P.O. Box 616, Maastricht 6200 MD, The Netherlands; Department of Hospitals Accreditation, Saudi Central Board for Accreditation of Healthcare Institutions, P.O. Box 2415, Riyadh 12264, Riyadh, Saudi Arabia; Department of Health Services Research, CAPHRI, Maastricht University Medical Center, Faculty of Health, Medicine and Life Sciences, Maastricht University, P.O. Box 616, Maastricht 6200 MD, The Netherlands; Department of Health Services Research, CAPHRI, Maastricht University Medical Center, Faculty of Health, Medicine and Life Sciences, Maastricht University, P.O. Box 616, Maastricht 6200 MD, The Netherlands

**Keywords:** accreditation, hospitals, healthcare quality, attitude, normalisation process theory

## Abstract

**Background:**

Accreditation is an important performance management tool. The engagement of stakeholders in pursuing accreditation plays a critical role in integrating standards into routine practice.

**Objective:**

This study explores the attitude of hospital directors towards accreditation and investigates the mechanisms of normalising standards in Saudi Arabian hospitals.

**Methods:**

Fifteen hospital directors across Saudi Arabia participated in semi-structured qualitative interviews. The interviews were conducted virtually, audiotaped, transcribed verbatim, and then analysed thematically using the NVivo-12 software package. The normalisation process theory (i.e. coherence, participation, actions and monitoring) was adopted to frame the study and describe the findings on normalising accreditation standards heuristically.

**Results:**

Overall, the hospital directors perceived accreditation favourably, particularly by those with more experience or previous exposure to accreditation. This attitude was a factor in normalising standards into daily operations. The clarity of standards, availability of full-time quality professionals and alignment of accreditation standards with hospital strategies assisted hospital directors in making sense of accreditation (coherence) and moving towards engaging hospital teams in the process (cognitive participation). This motivation-driven engagement catalysed the initiation of purposeful operational activities to integrate standards in operations (collective actions). The integration included distributing standard sets to relevant owners, conducting gap analysis, constructing a corrective plan and prioritising tasks within timeframes. Despite the financial and structural constraints experienced, the integration resulted in enhanced organisational safety culture, team spirit, communication, public trust, reporting of safety concerns and standardising of procedures. Following the integration, the objective appraisal of accreditation benefits (reflexive monitoring) was critical in addressing what went wrong, what worked well, and subsequently in sustaining performance gains.

**Conclusion:**

The effectiveness of integrating accreditation standards heavily relies on making sense of accreditation and understanding the mechanisms through which standards are routinised into operations. This study, using normalisation process theory constructs, indicates that standards integration phases are sequential, interlinked and influenced by culture, teamwork and leadership engagement. The findings helped in clarifying the accreditation operating process which may provide advantages to policymakers and stakeholders in making informed decisions on the implementation of accreditation.

## Background

Quality improvement is a strategic priority for all healthcare systems. Globally, accreditation has acquired a progressive position among quality improvement strategies. Accreditation is described as an external evaluation of healthcare institutions’ compliance with predefined standards [1]. A recent review emphasised that introducing accreditation as a stand-alone quality improvement solution might not guarantee outcomes and present stakeholders with an inflated impression of accreditation effectiveness [2]. The contextual heterogeneity of accreditation policies, scarcity of persuasive causal studies on its value, and the substantial expenditures necessary to meet accreditation standards could, in part, contribute to the conflicting views on the value of accreditation.

Similarly, the evidence on the perception of stakeholders on whether accreditation is effective presents a mixed picture [3]. Some studies criticise accreditation for being disruptive to patient care, timely, costly, bureaucratic and insensitive to outcomes [4, 5], while others praise its role in promoting organisational performance and standardising processes [6, 7]. Mitchell *et al*. [8] intensify the role of accreditation in innervating performance improvement by bridging the know-do gap. Indeed, evaluating the effectiveness of accreditation is heavily reliant on understanding the mechanisms through which standards are integrated into business operations.

Integrating standards in healthcare facilities is context-sensitive and is determined by diverse factors. In this context, the engagement of leaders in pursuing accreditation is one of the key determinants [9, 10]. Therefore, analysing how leaders perceive accreditation may contribute to fostering a greater acceptance and tailoring of accreditation design to hospital needs, thereby offering a deeper understanding of the mechanisms of normalising (i.e. making variable performance conform to standard) accreditation standards [11]. To address these issues, this study presents evidence on hospital directors’ attitudes towards normalising accreditation standards in Saudi Arabia.

In Saudi Arabia, there are over 450 public and private hospitals. The Ministry of Health is the major player in this system. The system currently is in a transformation towards patient-centric value-based healthcare. Alongside other management tools, a mandatory accreditation scheme to enhance the quality of healthcare services has been adopted. The Saudi Central Board for Accreditation of Healthcare Institutions (CBAHI) is the authorised accrediting entity that sets and evaluates the compliance of hospitals with performance standards. The CBAHI accreditation pathway is well-defined based on the International Society for Quality in Healthcare standards. Following an onsite survey, a hospital is granted an accreditation certificate valid for three years if the compliance level matches pre-established criteria. Yet, there have been no studies on the working process of accreditation or the attitudes of hospital leaders towards accreditation in the Saudi context.

The aim of this study is 2-fold; first, to explore the attitude of hospital directors towards Saudi Arabia’s national accreditation programme, and second, to investigate the mechanisms through which accreditation standards are normalised in hospital operations, using the normalisation process theory (NPT), which is a sociological middle-range theory that offers heuristic explanations for the mechanism of incorporating complex interventions, such as accreditation, into routine practice [12].

## Methods

### Design and sample

In concordant with the exploratory nature of the study, a semi-structured qualitative interview method was employed to rigorously explore the research aims. Since exposure to recurring accreditation visits might influence the perception of hospital directors and hence jeopardise the validity of our findings [13], the inclusion was limited to hospitals that had had one accreditation visit and had subsequently been accredited for at least six months prior to the interview. The publicly accessible list of accredited hospitals on the CBAHI website revealed that 20 hospitals satisfied our inclusion criteria [14]. The leading individuals in these hospitals (called henceforth ‘hospital directors’) were invited to participate in the study, provided that they had been in their positions for at least six months prior to the accreditation visit and six months thereafter. Consistent with previous studies [15, 16], this timeframe was assumed to be sufficient for them to acquire adequate exposure and an understanding of the accreditation processes. Of the 20 hospital directors approached, two did not meet our timeline criteria, while three declined participation for personal reasons. A consent form and an explanatory information sheet were emailed to the remaining 15 participants. Consent was deemed to have been declared if the email was replied to with a positive response. Next, one-to-one interviews were scheduled for times that suited the participants.

### Qualitative interviews and transcript preparation

All the interviews were conducted and recorded virtually by the main researcher (MH), using the Zoom videoconferencing platform, during the period May to June 2021. The security and cost-effectiveness of virtual qualitative interviews have been praised, particularly when participants are geographically dispersed [17]. At the commencement of the interviews, consent declarations were verified and voluntary participation was emphasised. The interviews were then directed using an interview guide that had been meticulously developed by the research team following an extensive review of the existing literature. The guide featured a series of open-ended questions that were informed by the NPT to reveal various aspects of the implementation of accreditation (see Supplementary A). Additionally, probing questions were used to assist in clarifying potentially confusing aspects. No new information emerged after 12 interviews, which was further confirmed when the remaining three interviews were completed, indicating thematic saturation and sample size adequacy [18]. On average, each interview lasted for 40 minutes. Thereafter, the interviewer transcribed the audiotapes verbatim and shared the transcriptions with the participants at the earliest possible time for comments and corrections [19].

### Transcript analysis and theoretical framework

The main researcher reviewed the transcriptions to get acquainted with the data and detect suitable codes. Thematic content analysis was employed to aggregate similar textual segments into a single code, and then group the interlinked codes into a relevant theme [20]. Subsequently, multiple thematic refinements were assumed to avert overlapping and to ensure the logical grouping of identified themes. Notably, the NPT was adopted as an explorative model to elucidate the working mechanisms of accreditation, from introduction to normalisation [12]. The theory distinguishes between four integrated constructs that focus on the work required to accomplish routinisation (coherence, cognitive participation, collective actions and reflexive monitoring), which offers a rigorous analytical framework to understand the dynamics influencing the successful deployment and integration of a new intervention, such as accreditation, into routine practice. Hence, we determined the suitability of NPT to characterise the dynamic actions required by stakeholders to integrate accreditation standards into business operations. Consequently, emerging themes were sorted taxonomically under the constructs outlined in the NPT. The NVivo-12 software package was used to structure the iterative codes. An illustrative coding tree is presented in Supplementary B.

### Qualitative trustworthiness and reporting

To ensure the trustworthiness of our study, numerous credibility, transferability and dependability endorsements were employed. Measures such as testing the efficiency of the interview guide, allocating sufficient time to collect data, iterative questioning, constant peer debriefings, member checking and theoretical guided analysis were used to ensure credibility. Additionally, methodical coding verification, reaching thematic saturation and carrying out the study protocol as initially planned were deemed necessary to ensure the transferability of the findings to other contexts. Furthermore, the Consolidated Criteria for Reporting Qualitative Research (COREQ) checklist was assumed to assure dependability, improve reporting quality and facilitate the derivation of intelligible and auditable conclusions [21]. The findings were supported by transparent, yet anonymous, quotes. The participants were designated by the letter ‘P’, followed by an Arabic numeral denoting the order of an interview.

## Results

In total, 15 hospital directors were interviewed, most of whom were physicians with over six years of experience. Approximately half of them (47%) had been in their current posts for three years or less (Table 1). The hospitals had been surveyed for accreditation between July 2019 and October 2020. Most of them were public (60%), provided acute care service (73%) and had less than 300 beds (86%). On average, these hospitals employed a full-time quality professional for every 25 to 30 beds.

**Table 1:** Demographics of participants (n = 15)

Characteristics	n (%)
Gender, male	15 (100)
Educational background	
Physicians	8 (53)
Health Administration	4 (27)
Others	3 (20)
Level of education	
Bachelor	9 (60)
Master	5 (33)
PhD	1 (7)
Total years of experience	
4–6 years	3 (20)
7–9 years	5 (33)
>9 years	7 (47)
Experience in the current position	
1–3 years	7 (47)
4–6 years	5 (33)
>9 years	3 (20)
Previous experience in accreditation	
Yes	7 (47)
No	8 (53)

Thematically, a total of 621 textual segments were grouped into 29 codes, which were subsequently used to synthesise 11 distinct, yet interrelated, themes. Despite disparities in participant and hospital characteristics, no thematic differences were identified. The emerging themes were tabulated into the NPT constructs; coherence, cognitive participation, collective actions and reflexive monitoring, as summarised in Table 2.

**Table 2:** Summary of themes and codes from the participant’s perspective

NPT Constructs	Themes	Codes
Coherence: How hospital directors understand and recognise hospital accreditation programmes individually and collectively.	Sense-making towards the accreditation program	Define accreditation Understand accreditation processes Recognise the anticipated benefits of accreditation
	Understand accreditation standards	Standards clarity and relevancy Alignment of accreditation standards with organisational strategic goals
Cognitive participation: How hospital directors sustain engagement in accreditation implementation individually and collectively.	Attitude	Perceiving change positively Sceptical approach towards change
	Time consumption	Time required for initiation and integration.
	Organisational engagement	The role of leaders in driving accreditation Motives for leaders’ participation Motives for team participation
Collective actions: How hospital directors integrate accreditation standards with daily business operations.	Integration and operationalisation	Gap analysis and taskforces formation Enact a set of implementation practices Monitoring the progress of implementation
	Resources allocation	Direct financial expenditure Indirect financial expenditure
	Workability	Driving factors of accreditation implementation Restraining factors of accreditation implementation
Reflexive monitoring: How do hospital directors reflect and appraise the accreditation programme and its effect?	Appraise accreditation programme	Appraise surveying activities Appraise surveyors (i.e. evaluation team)
	Evaluate effectiveness & worthiness	Impact at the organisational level Impact at the patient level Impact at the staff level Impact on the clinical outcomes Impact on the economic outcomes
	Practice differently	Moving towards patient-centeredness Deploy quality and patient safety culture Utilise team-based approach Embrace performance management and benchmarking

NPT, normalisation process theory.

### Coherence

Responses on defining accreditation were heterogeneous and influenced by various determinants. The participants with fewer years of experience described accreditation as an evaluation tool to detect system insufficiencies or a marketing tool to enhance reputation, whereas those with longer experience or who had had previous exposure to accreditation processes defined accreditation as a management tool that assisted in outlining business activities and promoting the quality of care. One of the participants commented:

‘I am the hospital director today but a patient tomorrow. Quality improvement in the target, while accreditation is a supporting tool that stimulates the process of implementing quality systems’ (P12)

Four primary concerns were raised by the participants when initially faced with the accreditation programme: the mandatory nature, the irrelevancy of the standards in specialised hospitals, the large proportion of professionals with limited quality literacy, and a lack of quality culture. However, the participants emphasised the role of the clarity of standards, the availability of full-time quality professionals, and the alignment of accreditation standards with hospital strategic plans, in accelerating the coherence phase towards engaging hospital teams in the process. As stated by one of the participants:

‘I think, obligating accreditation might defeat its purpose and give the process an inspection flavour […], it contradicts the commitment to duty of the health professionals toward patients’ (P4)

### Cognitive participation

All the participants consistently underlined the important role of hospital directors in driving the implementation of accreditation. Alike, they emphasised the necessity for teamwork and engagement of frontline staff in coproduction. The analysis revealed two management approaches in terms of engagement. Following the first approach, most participants perceived the capacity of accreditation to promote the practice positively. Consequently, they eagerly assumed administrative and technical roles in leading the change. The main motivators in this approach were dedication to safety, meeting strategic goals, enhancing the learning experience and raising external reputation.

In the second approach, the participants adopted a delegative style of quality-related activities and tended to acquire accreditation with the least possible effort. The participants ascribed this sceptical approach to the lengthy accreditation process, the reluctance of health workers to participate and the magnitude of anticipated changes. The main grounds for participation were marketing, regulatory obligations and pressure from governance bodies. As one participant put it:

‘each standard represents a level of quality to be attained, and each attainment requires introducing certain changes whether on small or hospital-wide scales. I was not ready to begin this experience while surrounded by hesitant co-workers’ (P7)

Several strategies, such as involving staff in the design phase, incentives, awareness campaigns, maintaining quality as a standing agenda item in departmental meetings, and presenting standards alongside convincing factual evidence (i.e. empirical-rational strategy), were employed to facilitate the engagement of frontline staff in the change process. This engagement was the paramount catalyst for moving into the action phase, as illustrated in the following quote:

‘the most often asked question along the way was “why is this standard important?” supporting the explanation with evidence was the secret buy-in strategy to get everybody on-board and kick-off implementation, particularly healthcare professionals’ (P12)

### Collective actions

The participants employed a bundle of purposeful operational activities to integrate standards into daily operations. Initially, standard sets were distributed to relevant owners to familiarise them with the content. Besides, task forces were formed to undertake gap analysis, construct a corrective plan, prioritise tasks and define timeframes accordingly. Concurrently, communication and monitoring systems were established to enhance efficiency, encourage relational work between and within taskforces and ensure prompt implementation of actions. Subsequently, tasks such as policy development, infrastructure repair and training were initiated. Occasionally, due to time constraints, certain activities were patchy, or improperly implemented (i.e. workarounds), to comply with the standards. This premise can be seen in the following extract:

‘the required time considerably surpassed our estimates and plans. We tend to use shortcuts as we were in a race against the clock, and we postponed determining what went wrong until after the survey visit’ (P1)

The integration process was influenced by multiple factors. The main challenges addressed by the participants were financial constraints, workforce insufficiency and infrastructural inadequacy. Nonetheless, as described by the participants, adequate support for the process and taskforces at this point was vital in attaining accreditation, despite hurdles and pressures. One of the participants summarised the challenges by saying:

‘accreditation process was not without cost. In addition to the direct expenses such as manpower recruitment and training. An indirect cost was demonstrated by pulling our health professionals away from their clinical duties’ (P14)

### Reflexive monitoring

Most participants agreed that an objective evaluation of accreditation worthiness following the integration of standards was critical to understanding new practices, averting the undermining of accreditation effectiveness and sustaining performance gains. The evaluation included surveying activities, revisiting the performed time-saving shortcuts and identifying residual nonconformance, thus gleaning lessons from the achieved successes.

Overall, the participants viewed accreditation favourably. As described, the integration of standards was associated with the adjustment of various internal practices related to patient-centeredness, safety and performance management. This enhanced organisational safety culture, as evidenced by the creation of a common quality language among staff. In addition, fostering team spirit, enhancing communication, standardising procedures, public trust and increasing the reporting of safety concerns were delineated. These effects were attributed to preparatory efforts rather than the accreditation visit itself. Noteworthily, although the participants reported no unintended consequences associated with the process other than co-worker stress, several reflective concerns were raised regarding variability among surveyors, the reliability of evaluating performance using a snapshot sample and the capacity of accreditation to produce sustainable patient and economic outcomes. One participant stated:

‘I have seen processes such as outpatient waiting time, cancellation rate in the operating room, and hand hygiene compliance improved considerably. However, I cannot presume an impact on patient outcomes following the survey, probably more time is needed to determine that’ (P8)

## Discussion

### Statement of principal findings

This qualitative study found that hospital directors, particularly those with more experience or previous exposure to accreditation, viewed accreditation favourably. Indeed, several factors assisted hospital directors in making sense of accreditation and initiating multiple mechanisms to normalise standards into business operations subsequently. In our study, the NPT constructs outlined these normalisation mechanisms. Importantly, the normalisation resulted in enhanced organisational safety culture, team spirit, communication, public trust, reporting of safety concerns and standardising of procedures.

### Strengths and limitations

To our knowledge, this is the first study to explore the attitudes of hospital directors towards accreditation and to investigate the mechanisms through which accreditation standards are normalised in Saudi Arabian hospitals. Although our results are relevant to a broad context, the transferability of the results should still account for contextual differences. As to limitations, the inherent recall bias of qualitative studies may have biased our results. However, adopting a theoretical framework, employing trustworthiness techniques, reaching thematic saturation, and using methodological coding increased the credibility of our findings and assisted in structuring a conclusion that is highly consistent with accreditation publications. In the analysis, due to the known overlap across the NPT constructs [12], themes were allocated meticulously in a mutually exclusive manner to avert possible duplication and reserve the inductive nature of the study.

### Interpretation within the context of the wider literature

Consistent with previous studies [6, 10, 22], the overall attitude of the participants towards accreditation was favourable. However, the years of experience might have had a confounding effect, as those participants with longer experience or previous exposure to accreditation perceived accreditation more meaningfully; a notion that is supported in Ellis *et al.*’s study [9].

In alignment with the NPT framework, our findings indicate that the implementation phases of accreditation are sequential and interlinked. The progress in each stage is highly influenced by culture, teamwork and the degree to which hospital directors understand and orchestrate accreditation, as described by the participants. In our study, making sense of the accreditation programme and standards, ‘coherence’, greatly affected participants’ attitudes towards assuming a leading position in the implementation process. Although the participants were, hierarchically, in an influential position, the cultural resistance to introducing a major change during standards integration requires a blurring of the line between leaders and frontline workers—‘cognitive participation’. This collective engagement lends credence to previous studies that emphasised the crucial role of teamwork in implementing complex interventions [23, 24]. However, engaging frontline workers was a strenuous task that required individualised approaches to be successful.

In the implementation phase, ‘collective actions’, a series of purposeful activities, were necessary to routinise standards in daily operations. As reported in various contexts, these actions were challenged by financial restrictions [4, 25], structural inadequacy and sceptical behaviour of leaders [6]. Furthermore, time constraints and co-worker stress generated certain workarounds to achieve artificial happy ends, resulting in a mismatch between the actual practice and the evidence handed to the accreditation survey team [24, 26]. The reported stress reaffirmed the need for suitable protocols to support co-workers throughout accreditation. Last, the post-survey appraisal was used to address what went wrong and what worked well—‘reflexive monitoring’. In agreement with prior studies [7, 25, 27, 28], the oft-reported positive effect was the promotion of patient safety culture. However, several concerns were raised by the participants, such as variability among surveyors [22], the irrelevance of some standards [10] and the uncertainty of outcomes [29]. The latter may be, in part, attributed to the nature of accreditation standards that emphasise organisational structure and process rather than outcomes [30].

### Implications for policy, practice and research

Our findings emphasise the importance of exploring the attitude of hospital directors in developing and implementing accreditation schemes. Failure to engage stakeholders in the process may result in disillusionment and alienation from the accreditation. We echo recent publications urging accrediting agencies to adopt a bottom-up approach in designing and flowcharting the accreditation process [9, 11, 23, 26]. Despite cultural differences, the contextual lessons learnt from this study offer stakeholders and policymakers evidence to assist them in implementing and evaluating accreditation effectively and are anticipated to demonstrate implications that cross boundaries due to the high degree of similarity in accreditation programmes worldwide [1]. Future studies, which might be based on NPT, are necessary to evaluate the strategies that consolidate the engagement of stakeholders. Furthermore, a longitudinal investigation of changes in the attitudes of leaders towards accreditation over recurrent accreditation cycles may also add value.

## Conclusion

Exploring the attitudes of hospital directors towards accreditation reveals aspects that influence the integration of accreditation standards and contribute to the long-term sustainability of accreditation programmes. The effectiveness of integrating accreditation standards heavily relies on making sense of accreditation and understanding the mechanisms through which standards are routinised into operations. This study found that hospital directors perceived accreditation favourably. Using NPT constructs, the results also indicate that standards integration phases are sequential, interlinked and influenced by culture, teamwork and leadership engagement. The findings help in clarifying the accreditation operating process, which may be helpful to policymakers and stakeholders in making informed decisions on the implementation of accreditation.

## Supplementary Material

mzac070_Supp

## Data Availability

Data underlying the findings of this study are available within the article [and/or] its supplemental materials. Additional data will be shared at reasonable request to the corresponding author in a de-identified form for confidentiality purposes.
