# Supplementary material for: The attitudes of hospital directors towards normalising accreditation standards: A qualitative descriptive study for Saudi Arabia
Source: Int J Qual Health Care. 2022 Sep 1;34(3):mzac070. doi: 10.1093/intqhc/mzac070 (PMC9470101; doi:10.1093/intqhc/mzac070)
Supplement: mzac070_Supp [file mzac070_supp.zip › Supplement A - Interview Guide.pdf]

Supplement A: Interview Guide

SECTION I: HOSPITAL CHARACTERISTICS | SECTION II: PARTICIPANT DEMOGRAPHICS

Items	Items
Hospital sector	Educational background
Type of hospital (i.e., specialization)	Level of education
Total number of active beds	Gender
Name of accrediting body & number of cycles	Job Title
Accredited? If yes, total years of being accredited	Total years of experience
Date of the last accreditation visit	Years of experience in the current position
Number of quality management staff in the hospital	

SECTION III: OPEN-ENDED QUESTIONS

Coherence / Sense-Making	
**Question 1:**	**Would you tell me about your accreditation experience?**<br>**Probing Questions:**<br>- In how many accreditation cycles have you participated in your experience?<br>- What hospital accreditation programs have you participated in?
**Question 2:**	**What is the purpose of accreditation from your perspective?**<br>**Probing Questions:**<br>- Is the accreditation process aligned with strategic priorities and goals in your hospital?<br>- What are the potential values anticipated from participating in accreditation programs?
**Question 3:**	**Did accreditation affect the nature of your work?**<br>**Probing Questions:**<br>- How useful did you find accreditation standards in doing things different than usual?<br>- How would you perceive accreditation if it is an optional program?
**Cognitive Participation / Engagement**	
**Question 4:**	**Would you describe your role in the accreditation journey at your hospital?**<br>**Probing Questions:**<br>- How would you describe your approach to working with accreditation tasks?<br>- At a personal level, what was the main motivator for your participation in accreditation?

**Question 5:**	**How much time has your hospital spent in understanding and working on accreditation standards at the preparatory phase?**<br>**Probing Questions:**<br>- How do you perceive the clarity and the focus of accreditation standards?
**Question 6:**	**How do you describe the engagement of your hospital in internal and external accreditation activities?**<br>**Probing Questions:**<br>- Describe internal (e.g., individually) and external (e.g., stakeholders) engagements with accreditation activities?<br>- What factors encouraged employee engagement in the accreditation journey at your hospital?
**Collective Action / Implementation**	
**Question 7:**	**What task have you had during working on meeting accreditation standards?**<br>**Probing Questions:**<br>- How do you describe your participation level in accreditation standards implementation?
**Question 8:**	**What interventions/actions have been taken in your hospital to implement accreditation standards?**<br>**Probing Questions:**<br>- Was additional training or external consultation needed during accreditation implementation?<br>- How accreditation preparation tasks were allocated to various departments, units, & individuals?
**Reflexive monitoring / Appraisal**	
**Question 9:**	**How do you think accreditation affected the quality of service in your hospital?**<br>**Probing Questions:**<br>- How accreditation has affected your hospital at organizational, customer, staff, and outcome levels?<br>- Did you experience any unintended consequences of the accreditation process?<br>- What values were you anticipating from the accreditation survey that did not come true?
**Question 10:**	**How do you perceive the process of the on-site accreditation visit?**<br>**Probing Questions:**<br>- How do you perceive the accreditation evaluation process?<br>- How do you perceive the accreditation surveyor's reliability?<br>- To which extent accreditation standards are clear and relevant to your hospital setting?
**CLOSING**	**Is there anything else you would like me to know?**
