# Supplementary figures and images for: The attitudes of hospital directors towards normalising accreditation standards: A qualitative descriptive study for Saudi Arabia
Source: Int J Qual Health Care. 2022 Sep 1;34(3):mzac070. doi: 10.1093/intqhc/mzac070 (PMC9470101; doi:10.1093/intqhc/mzac070)

## Supplement B: Coding Tree

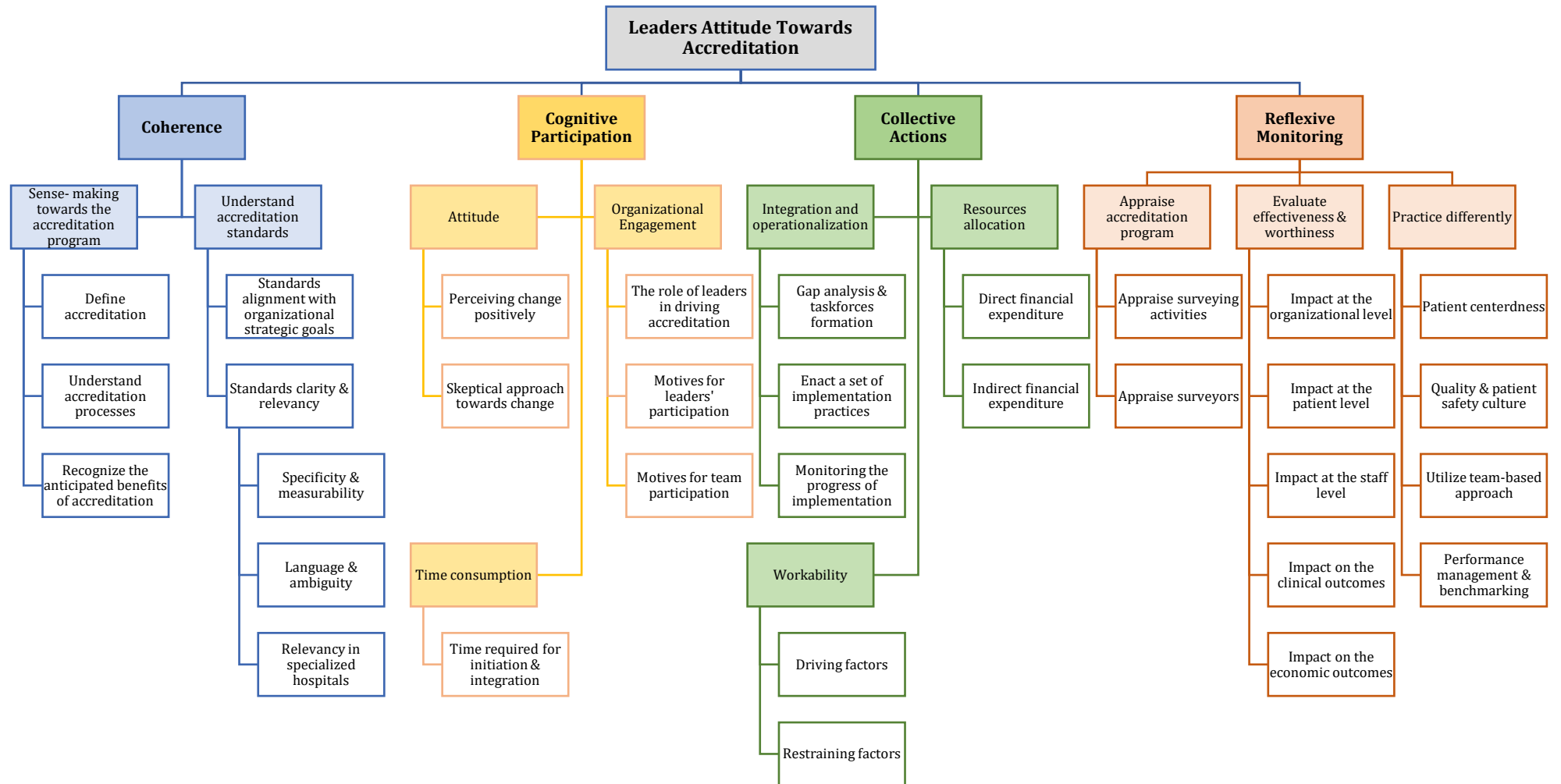

Supplement: mzac070_Supp [file mzac070_supp.zip › Supplement B - Coding Tree.pdf]
